# Thoracic aggressive vertebral hemangioma with neurologic deficit: A retrospective cohort study

**DOI:** 10.1097/MD.0000000000012775

**Published:** 2018-10-12

**Authors:** Wei Hu, Shun-Li Kan, Hui-Bin Xu, Ze-Gang Cao, Xue-Li Zhang, Ru-Sen Zhu

**Affiliations:** a Department of Spine Surgery, Tianjin Union Medical Center; b Department of Orthopaedics, Wuqing TCM Hospital affiliated to Tianjin TCM University, Tianjin, China.

**Keywords:** neurologic deficit, percutaneous curved vertebroplasty, thoracic aggressive vertebral hemangioma

## Abstract

The aim of this study is to evaluate the safety and effectiveness in the treatment of thoracic aggressive vertebral hemangiomas (AVHs) with neurologic deficit by multiple surgical treatments.

The clinical and radiographic data of 5 patients suffering from thoracic AVHs with neurologic deficit and treated by multiple surgical treatments, including percutaneous curved vertebroplasty (PCVP) combined with pedicle screw fixation and decompressive laminectomy, were reviewed and analyzed retrospectively.

Five patients (3 women and 2 man, with a mean age of 57.40 ± 11.93) were diagnosed with AVHs from July 2010 to April 2016. All of them had objective neurologic deficit, myelopathy, and back pain. They underwent multiple surgical treatments and were followed-up for 12 to 23 months. At final follow-up, Frankel Grade D was achieved in all 5 patients. Patients were free from pain and neurologic symptoms, and the functional status was improved. No major complication was found.

The treatment of AVHs with neurologic deficit is a challenge for surgeons. PCVP combined with pedicle screw fixation and decompressive laminectomy is safe and effective, and can be used for AVHs with neurologic deficit. Further studies with more samples are required to validate the effectiveness and safety of PCVP combined with pedicle screw fixation and decompressive laminectomy.

## 1 Introduction

Vertebral hemangiomas (VHs) are benign vascular neoplasms in the vertebral column and often found in the thoracic and lumbar spine. They are the most common vertebral tumors with an incidence of 10% to 12% in the spinal autopsies, are usually asymptomatic, and are discovered accidentally.^[1,2]^ In most of patients, these lesions typically manifest asymptomatic and do not necessitate any special intervention. In very few patients, however, aggressive vertebral hemangiomas (AVHs) can cause local pain, radicular pain, or neurologic deficits, which result from neural compression caused by bone expansion, erosion through cortex, fracture, or hematoma.^[3–5]^ There were large numbers of interventions^[6–9]^ which have been employed in the treatment of AVHs with neurologic deficit. But there is controversy for the treatment of AVHs with neurologic deficit. For patients with mild to moderate pain, conservative treatment can relieve their symptoms. However, if the symptoms of patients are neurologic deficits and intractable pain, only surgical and nonsurgical intervention can be chosen to relieve those symptoms.^[10]^ Percutaneous curved vertebroplasty (PCVP) with polymethyl methacrylate injection was regarded as a relatively new, palliative technique for the treatment of AVHs.

In this study, we reported PCVP combined with pedicle screw fixation and decompressive laminectomy treatment and follow-up of 5 patients with AVHs. The objective of these techniques was to minimize invasive, reduce the rate of complication, and relieve pain rapidly.

## 2 Materials and methods

### 2.1 Patient information

From July 2010 to April 2016, 5 consecutive adult patients suffering from symptomatic AVH with epidural extension underwent mixed surgery, which included pedicle screw fixation, vertebroplasty, and decompressive laminectomy.

A retrospective review of clinical medical history and radiographs was performed. This study was approved by the Institutional Ethical Review Board of Tianjin Union Medical Center.

### 2.2 Imaging study and examination

Anteroposterior and lateral spinal X-rays, computed tomography (CT), and magnetic resonance imaging (MRI) were customarily implemented for all of the patients. Preoperative MRI and CT revealed the typical pattern of spinal cord compression because of AVH.

Clinical examination was performed using the visual analog scale (VAS) and the Japanese Orthopaedic Association (JOA) scale. Neurologic examination was carried out with assessment of sensory symptom, motor deficits, and Frankel grade.

### 2.3 Surgical technique

**Figure 1:**
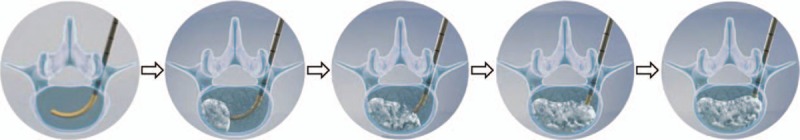
Description the application of the percutaneous curved vertebroplasty.

All procedures were carried out under general anesthesia and carefully monitored. Under the guidance of C-arm, the incision site was marked above the affected vertebra. Afterwards the field of operation was exposed. Pedicle screws were fixed above and below the affected vertebral body firstly. A puncture needle was placed percutaneously into the posterior vertebral body using a unilateral transpedicular approach. The curved probe (Ningbo Hicren Biotechnology Co, Ltd, Ningbo, China) was placed into the vertebral body through the cannula of the needle (Fig. 1). The bone cement was injected close to the anterior border of the vertebral body. About 3 to 4.5 mL of bone cement was injected. It was necessary to fill-up the lesion and the entire vertebral body without any bone cement leakage to destroy and shrink the malformation. Then direct lesion decompression was implemented to decompress and visualize the dura after the laminas over the lesion were removed. Intraoperative biopsies were performed for histologic test in the 5 patients.

### 2.4 Follow-up

We got X-rays at 3-, 6-, and 12-month follow-up and annually. A CT scan was carried out at the 3-month follow-up and repeated annually in all patients. Neurologic status was recorded from presentation to outpatient visits. The Frankel grade was utilized to evaluate the level of spinal cord injury preoperatively and postoperatively. Each patient was evaluated through subjective perception and functional status.

### 2.5 Statistical analysis

Continuous variables are expressed as mean ± standard deviations. The *t* test was used to evaluate the significance of changes from preoperative to postoperative in the VAS and JOA. A *P*-value <.05 was considered statistically significant. All statistical analyses were carried out with SPSS 17 software (SPSS Inc, Chicago, IL).

## 3 Results

### 3.1 General results

There were 5 patients in this study, 3 women and 2 man, with a mean age of 57.40 ± 11.93 (range: 44–75 years), and mean follow-up of 17.60 months (range: 12–23 months) (Table 1). All 5 patients exhibited with objective neurologic deficit owing to VH. The patients experienced mild to moderate back pain, tingling sensation, numbness, and weakness of lower limbs. All of them suffered from myelopathy, including 2 cases with Frankel Grade B, 2 with Frankel Grade C, and 1 with Frankel Grade D. At baseline, all patients had back pain with a VAS ≥5 (mean VAS 6.80 ± 1.10). Neurologic function examination indicated that 2 patients presented with positive Babinski sign.

**Table 1:**
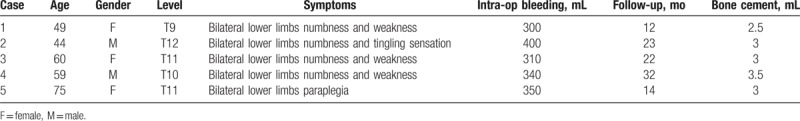
Population characteristics.

### 3.2 Clinical improvement

All patients relieved instantly from pain and neurologic symptoms, and were discharged on the 5th day after surgery. All patients recuperated adequately to return to work. At final follow-up, Frankel Grade D was achieved in all 5 patients with Frankel Grade B, C, or D, and all of them were released from pain (mean VAS 2.60 ± 0.55) (*P* < .05) and neurologic deficit. The JOA scores ranged from 6.2 ± 0.84 (preoperation) to 13.4 ± 0.55 (postoperation) (*P* < .05).

### 3.3 Radiographic improvement

All patients triumphantly underwent surgery to decompress the compressed spinal cord. The intraoperative imaging demonstrated the damaged vertebra was completely filled with bone cement and well distributed. Bone cement leakage was found in 1 patient. The minor bone cement leaked out at one side of the vertebral body and did not have any clinical influence. No bone cement leaked into the spinal canal. Cement pulmonary embolism was not found. The amount for bone cement implanted per affected vertebra ranged between 2.5 and 3.5 mL.

### 3.4 Complications

**Figure 2:**
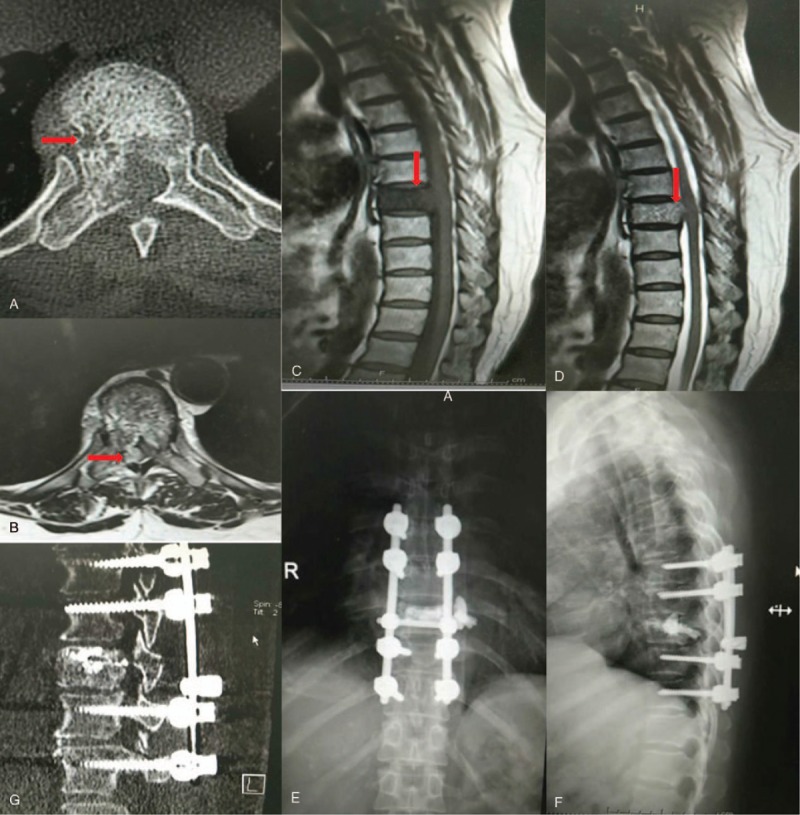
A 49-year-old female patient was diagnosed as having T9 whole vertebral hemangioma. (A) Axial computed tomography (CT) scan shows the “polka-dot” pattern of the hemangioma in T9 vertebral body (red arrow). (B) Axial magnetic resonance imaging shows ballooning of the posterior vertebral body wall and tumor, compressing the thecal sac (red arrow). (C, D) Low signal and high signal of the T9 vertebral body is seen in the T1 and T2 weighted image (red arrow), respectively. (E, F) X-ray 12 months after operation indicates that the height of T9 vertebral body was maintained and the location of pedicle screw is perfect. (G) Sagittal CT scan shows T9 vertebral body is filled with polymethyl methacrylate.

No surgery-related complications were found. Cement leakage was identified in a patient; however, it did not have an influence on the patient (Fig. 2).

### 3.5 Follow-up

The follow-up period ranged from 12 to 32 months. Patients got relief from pain and neurologic symptoms, had considerable improvement regarding functional status, and described slight or no residual pain in daily activities.

## 4 Discussion

The VH is a common clinical disease. A small number of patients with VH present with invasive, which sometimes compress the spinal cord, leading to neurologic deficit.^[11,12]^ Surgical interventions are necessary for patients with invasive VH accompanied with neurologic deficit. The diagnosis for this kind of patients is mainly based on imaging and postoperative pathologic examination results. In these patients, the typical performance in axial CT scans is “polka dot” or “round sign.” Moreover, the entire vertebral body and the related accessory structure are generally invaded by the lesion, resulting in cortical bone expanded and damaged.

Patients with AVHs may benefit from different interventions, such as depression, embolization, radiation therapy, and vertebroplasty. Vertebroplasty was regarded as an alternative treatment for AVHs. Vertebroplasty can stabilize the tiny fractures and prevent the vertebral body from further compression. Bone cement will produce a lot of heat, which inactivates tumor cells. In our cases, due to no loss of vertebral height in operation X-ray, PCVP was chosen. And all of them also showed strong indications for minimally invasive approach. The key of this technique is to fill the vertebral lesion with cement completely and make sure the sclerosis of the hemangiomatous venous pool cannot be reversible. Under the C-arm guidance, the needle is guided to go through the center of the pedicle and then into the vertebral body. The unipedicular approach was used, and the curved puncture needle entered through the intact pedicle. There were several reasons for us to do these: first, the anatomical structure of contralateral pedicle is normal, which may be conductive to the injection of bone cement and decrease the risk of cement leakage because the destruction of vertebral body may be associated with cement leakage^[13]^; second, if the lesion is malignant, puncturing the lesion may pollute the normal tissue and cause tumor metastasis; third, the unipedicular approach may ensure the bone cement was injected in the same period of cement coagulation, and decreased the risk of pulmonary embolism (PE); fourth, it may reduce the risk of PE; fifth, this approach may decrease the procedure time and increase the patients’ comfort.

The procedure of our surgery was divided into 3 steps: the first step was to fix the pedicle screws; the second step was to inject the bone cement. According to a previous study, one of the most common complications causing spinal cord compression or PE was cement leakage.^[14]^ The viscosity of bone cement was associated with bone cement leakage. A thin liquid would increase the risk of venous spread, and a thick mixture may be too viscous to inject, so the surgeons should be familiar with the polymerization time of bone cement.^[14]^ In the present study, the bone cement was injected when it reached the texture of a thickened cake glaze or paste, which may reduce the risk of bone cement leaking into the spinal canal effectively. The cement should be injected very slowly and carefully. Meanwhile, surgeons should pay attention to whether the bone cement leaks or not. In the absence of leakage, the maximum amount of bone cement should be filled into the vertebral body, and the general volume of cement injected range from 3 to 5 mL. The last step was to decompress. After bone cement was injected, the hemorrhage of the venous pool may be reduced, which may increase the clarity of the surgical area and may be contribute to decompress completely.

## 5 Conclusion

The treatment of AVHs with neurologic deficit is a challenge for surgeons. PCVP combined with pedicle screw fixation and decompressive laminectomy is safe and effective, and can be used for AVHs with neurologic deficit. Further studies with more samples are required to validate the effectiveness and safety of PCVP combined with pedicle screw fixation and decompressive laminectomy.

## Author contributions

**Conceptualization:** Wei Hu, Shun-Li Kan, Xue-Li Zhang, Ru-Sen Zhu.

**Data curation:** Wei Hu, Shun-Li Kan.

**Formal analysis:** Wei Hu, Shun-Li Kan, Hui-Bin Xu, Ze-Gang Cao.

**Funding acquisition:** Wei Hu, Ru-Sen Zhu.

**Writing – original draft:** Wei Hu, Shun-Li Kan.

**Software:** Hui-Bin Xu, Ze-Gang Cao.

**Visualization:** Hui-Bin Xu, Ze-Gang Cao.

**Project administration:** Xue-Li Zhang, Ru-Sen Zhu.

**Validation:** Xue-Li Zhang, Ru-Sen Zhu.

**Writing – review & editing:** Xue-Li Zhang, Ru-Sen Zhu.
